# Facilitation of Psychiatric Advance Directives by peer-workers: results from DAiP

**DOI:** 10.1192/j.eurpsy.2022.275

**Published:** 2022-09-01

**Authors:** A. Tinland, S. Loubiere, F. Mougeot

**Affiliations:** 1 APHM, Marss, Marseille, France; 2 APHM, Service Epidemiologie And économie De La Santé, Direction De La Recherche, MARSEILLE, France; 3 ENSEIS, Centre Max Weber, Lyon, France

**Keywords:** Advance decision making, Coercion reduction, Psychiatric advance directives, Peer workers

## Abstract

**Introduction:**

United Nations Convention on the Rights of Persons with Disabilities recognized that people with psychosocial disabilities have the same right to take decisions and make choices as other people. Consequently, direct or supported decision-making should be the norm and there should be no substitute decision-making. However, these principles are far from common practice in many mental health services. Joint-crisis plan (JCP) and Psychiatric advance directives (PAD) are interesting tools to translate the shared-decision making principle into clinical and practical reality. Most existing JCP or PAD involve facilitators, which improves their effectiveness, but facilitators are mostly professionals.

**Objectives:**

In this context, DAiP study was launched to evaluate the efficacy of PAD facilitated by peer-workers.

**Methods:**

DAiP was a multicenter randomized controlled trial conducted in 7 French mental health facilities, with a complementary qualitative approach. 394 adults with a DSM-5 diagnosis of schizophrenia (SCZ), bipolar I disorder (BP-I), or schizoaffective disorders (SCZaff ), who were compulsorily hospitalized in the past 12 months were enrolled from January 2019 and followed up for 12 months. Outcomes were compulsory admission rate, therapeutic alliance (4-PAS), quality of life (S-QOL), mental health symptoms (MCSI), empowerment (ES) and recovery (RAS).

**Results:**

In this communication, we propose to describe the practices of facilitation of peer-workers and analyze outcomes in lights of process measurements (whether or not participants completed PAD document, shared PAD and with whom, met facilitator, used PAD

**Conclusions:**

Involving peer-workers in the redaction of PADs coherently supports the current shift of mental health care from ‘substitute decision making’ to ‘supported decision making’.

**Disclosure:**

No significant relationships.

